# Beyond Financial Access: Socioeconomic Determinants of Health Engagement in Singapore’s Healthier SG

**DOI:** 10.1093/geroni/igaf122.3722

**Published:** 2025-12-31

**Authors:** Seoyeon Ahn, Alec Morton, Zhi Zhen Lim, Shumian Yeo, Cynthia Chen, Linda Wei Lin Tan, Xueling Sim, Ian Yi Han Ang

**Affiliations:** Duke-NUS Medical School/National University of Singapore, Singapore, Singapore; National University of Singapore, Singapore, Singapore; National University of Singapore, Singapore, Singapore; National University of Singapore, Singapore, Singapore; National University of Singapore, Singapore, Singapore; National University of Singapore, Singapore, Singapore

## Abstract

**Background:**

Singapore’s Healthier SG, launched in 2023, promotes voluntary primary care enrollment and participation in recommended screenings and vaccinations. Initially targeting adults aged sixty years and above, eligibility expanded to those aged forty years and above by 2025. This study examines how socioeconomic factors influence participation across engagement stages.

**Methods:**

We analyzed Singapore Population Health Studies data of adults aged forty years and above (N = 3,801) using multinomial logistic regression. Four Healthier SG engagement stages were examined: (1) unaware and unenrolled (reference), (2) aware but unenrolled, (3) enrolled but unscreened, and (4) enrolled and screened.

**Results:**

Older adults aged sixty years and above, those with more chronic conditions, and never-married individuals showed higher engagement rates. Compared to Chinese participants, Indians showed lower screening completion rates. Notable socioeconomic differences emerged: university education, homeownership, and non-labor force status significantly predicted being enrolled and screened. Marginal effects analysis revealed distinct age-related patterns—being enrolled and screened increased from 10.5% in the 40-49 age group to peak at 25.6% in the 60-69 age group, while program unawareness decreased sharply with age (59% to 45.7% respectively).

**Conclusions:**

Despite Singapore’s comprehensive preventive healthcare framework, engagement gaps persist across educational, housing, employment, ethnic, and age-related factors. This demonstrates that removing financial barriers alone is insufficient for achieving universal health access. Results underscore the need for targeted interventions addressing age, socioeconomic, and ethnic disparities through differentiated outreach, flexible implementation strategies, and culturally responsive approaches. This research provides actionable insights for designing inclusive health service delivery in diverse aging societies.

